# Not So Smooth Sailing: *FIG4* ‐Related Disease Is a Differential Diagnosis of Rapid Onset Dystonia‐Parkinsonism

**DOI:** 10.1002/mdc3.70049

**Published:** 2025-03-21

**Authors:** Matthew Julian Georgiades, Duncan Wilson, Maria Garcia, Robert Boland‐Freitas, Hugo Morales‐Briceño, Neil Mahant, Victor Shue Chung Fung, Andrew Martin

**Affiliations:** ^1^ Movement Disorders Unit, Department of Neurology Westmead Hospital Westmead NSW Australia; ^2^ Sydney Medical School University of Sydney Sydney NSW Australia; ^3^ Department of Neurology Blacktown Hospital Blacktown NSW Australia

**Keywords:** dystonia, parkinsonism, dystonia‐parkinsonism, *FIG4*, genetics

We report a patient with rapid onset progressive dystonia‐parkinsonism after minor head injury. The patient was found to have homozygous pathogenic variants in the *FIG*4 gene. The syndrome of rapid onset dystonia‐parkinsonism has not been described in association with *FIG4* variants, extending the phenotypic spectrum of homozygous *FIG4*‐related neurological disease and the genotypic differential diagnosis of rapid onset dystonia‐parkinsonism.

## Case Report

A 44‐year‐old right‐handed naval officer presented with acute‐onset, progressive gait disturbance, clumsiness, and insomnia. Symptoms began within 24 hours of sustaining a head strike while abseiling down a ship that was severe enough to cause a brief loss of consciousness, but no other immediate complications. His symptoms peaked to moderate severity within days, and then his disease continued to evolve over 18 months, with an asymmetrical jerky tremor and stiffness, worse on the left side, imbalance with frequent falls and marked speech impairment, obliging him to communicate primarily in gestures. He had neither autonomic, cognitive symptoms nor psychiatric symptom. He had neither significant medical history nor family history.

On examination (Video 1) there was hypomimia with reduced blink rate. The feet were high‐arched with hammer toes. Saccades were hypometric in all directions with normal latency and velocity; pursuit was normal. There were rare macrosaccadic oscillations in primary gaze. There was rightward truncal dystonia when seated, standing, and walking with left shoulder elevation. There was a predominantly left‐sided jerky, irregular and low‐amplitude resting and postural upper limb tremor. Mild upper limb rigidity was present with activation. There was severe, predominantly left‐sided limb bradykinesia with effortful rapid alternating hand movements and foot‐tapping. Writing was severely impaired. Postural reflexes were impaired. There were no pyramidal, sensory, or cerebellar signs. Speech was barely audible but sometimes improved when the patient gently pinched his throat. Laryngoscopy did not show adductor nor abductor dystonia but rather a marked delay in activation.

**Video 1:** The video demonstrates a jerky irregular, predominantly left‐sided tremor, along with asymmetric bradykinesia worse on the left side. There is rightward truncal dystonia in the seated position and during gait with a truncal tilt and left shoulder elevation. Infrequent macrosaccadic oscillations are observed in primary gaze. There is severe hypophonia with poor speech production that improves with a trick of placing the finger and thumb on the Adam's apple.

**FIG. 1:**
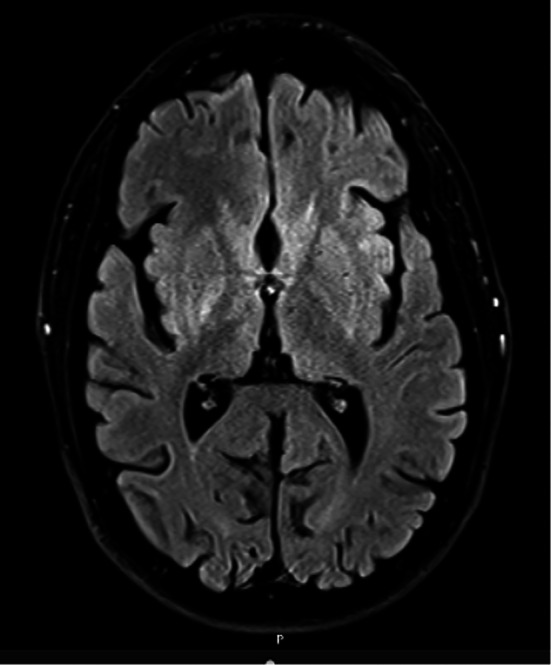
Axial T2‐FLAIR magnetic resonance imaging (MRI) of the brain showing asymmetric hyperintensity of the lentiform nuclei and thalamus. Additional sequences are available for review in the Supporting Information.

Routine investigations were normal. Cerebrospinal fluid showed mild protein elevation (0.77 g/L) but was otherwise normal. Magnetic resonance imaging (MRI) demonstrated basal ganglia and thalamic T2‐Fluid‐Attenuated Inversion Recovery (FLAIR) hyperintensities (Fig. 1). Susceptibility weighted imaging and T1 sequences were unremarkable (Supporting Information). Nerve conduction studies showed mild slowing of lower limb sensorimotor conduction. A low‐amplitude, irregular 7 Hz resting and postural tremor was demonstrated on neurophysiological studies. There was no myoclonus detected. Somatosensory‐evoked potentials suggested dysfunction of the large sensory fibers at the level of the nerve roots and/or dorsal root ganglia.

A syndromic diagnosis was made of rapid onset dystonia‐parkinsonism. Whole exome sequencing identified homozygous known pathogenic c.122 T > C(p.Ile41Thr) variants in the Factor Induced Gene 4 (*FIG4*); *ATP1A3* was normal. Levodopa (l‐dopa) up to a dose of 1000 mg/day was of no benefit, as was treatment with benztropine, trihexyphenidyl, amantadine, and clonazepam. The dystonia‐parkinsonism continued to worsen over the next 12 months with the development of gait freezing and more frequent falls with a component of sensory ataxia due to progressive sensorimotor neuropathy. At follow up (30 months after symptom onset) he also described mild subjective cognitive symptoms, worsening mood, and poor sleep.

## Discussion

We report rapidly progressive dystonia‐parkinsonism with onset after a head injury due to homozygous pathogenic variants in the *FIG4* gene.

*FIG4* encodes a phosphatase critical for intracellular vesicle trafficking along the endosomal‐lysosomal pathway.^1^, ^2^ *FIG4* variants produce diverse disease phenotypes depending on the degree of loss of protein function. Complete loss of function due to null alleles leads to Yunis‐Varón syndrome, a multi‐system disorder with severe neurological and skeletal developmental abnormalities that is often fatal in childhood.^3^ Partial loss of function mutations results in autosomal recessive Charcot–Marie‐Tooth (CMT) disease type‐4J, which is a neuropathy of variable severity and onset, but typically results in asymmetrical proximal muscle weakness unlike CMT disease, which classically causes distal weakness.^1^, ^4^ Biallelic compound heterozygous variants produce a continuum of overlapping syndromes consisting of slowly progressive parkinsonism with peripheral neuropathy that either predates the parkinsonism by many years or develops concurrently.^5^, ^6^ Parkinsonism has a variable response to l‐dopa.^5^, ^6^ Other described phenotypes include cerebellar ataxia, amyotrophic lateral sclerosis, and familial epilepsy with polymicrogyria.^7^, ^8^, ^9^ Rapid onset progressive dystonia‐parkinsonism has not been previously described.

Despite the phenotypic heterogeneity, symptom development in *FIG4‐*related disease is often triggered by physical trauma, and MRI may demonstrate thalamic and olivary T2 hyperintensities, as in our patient.^1^, ^6^, ^8^, ^9^ Nerve conduction studies typically demonstrate demyelinating features, which may be normal.^1^, ^4^

The syndrome of dystonia‐parkinsonism can be acquired (medication‐induced, lesion‐associated, toxic, infectious, prion, autoimmune) and genetic (*TAF1*, *PRKRA*, *ATP7B*, *GBA*, *HTT*, *SLC19A3*, *ATP1A3*, *KMT2B*, *PLA2G6*, *GCH1*, *PRNP*, and mitochondrial disorders—many of which are associated with iron accumulation).^10^ Apart from *ATP1A3* and prion disease, rapid onset or progression is not expected. *ATP1A3*‐related disease may evolve over hours to weeks often after physiological stress, then remain relatively stable with normal imaging.^10^ An *ATP1A3* variant was not detected in our patient.

Our patient extends the phenotypic spectrum of homozygous *FIG4*‐related neurological disease and the genotypic differential diagnosis of rapid onset dystonia‐parkinsonism.

## Author Roles

(1) Research project: A. Conception, B. Organization, C. Execution; (2) Statistical analysis: A. Design, B. Execution, C. Review and critique; (3) Manuscript preparation: A. Writing of the first draft, B. Review and critique.

M.J.G.: 1A, 1B, 3A, 3B

A.M.: 1A, 1B, 1C, 3B

D.W.: 1A, 1B, 1C, 3B

M.G.: 1A, 1B, 1C, 3B.

H.M.‐B.: 1A, 1B, 1C, 3B

N.M.: 1A, 1B, 1C, 3B

V.S.C.F.: 1A, 1B, 1C, 3B

## Disclosures

**Ethical Compliance Statement:** This work did not require ethics committee approval. The patient provided written informed consent. We confirm that we have read the journal's position on issues involved in ethical publication and affirm that this work is consistent with those guidelines.

**Funding Sources and Conflict of Interest:** No Specific funding was received for this work. The authors declare that there are no conflicts of interest relevant to this work.

**Financial Disclosures for the Previous 12 Months:** M.J.G. has no additional disclosures to report. D.W. received funding from the Edmond J Saffra Fellowship through The Michael J. Fox Foundation. N.M. has received honoraria from AbbVie, Medtronic, Merz, and Seqirus. V.S.C.F. receives a salary from NSW Health; has received unrestricted research grants from The Michael J Fox Foundation, AbbVie, and Merz; and receives royalties from Health Press Ltd and Taylor and Francis Group LLC.

## Supporting information


**Supplementary Figure S1.** (**A**) Axial T2‐FLAIR magnetic resonance imaging (MRI) of the brain showing asymmetric hyperintensity of the lentiform nuclei and thalamus. (**B**) T2, (**C**) susceptibility‐weighted, and (**D**) T1 sequences were unremarkable (images shown left to right).

## Data Availability

Data sharing is not applicable to this article as no new data were created or analyzed in this study.
