# Supplementary material for: Not So Smooth Sailing: FIG4 ‐Related Disease Is a Differential Diagnosis of Rapid Onset Dystonia‐Parkinsonism
Source: Mov Disord Clin Pract. 2025 Mar 21;12(Suppl 2):S10–2. doi: 10.1002/mdc3.70049 (PMC12372603; doi:10.1002/mdc3.70049)
Supplement: Supplementary file 1 — Supplementary Figure S1. (A) Axial T2‐FLAIR magnetic resonance imaging (MRI) of the brain showing asymmetric hyperintensity of the lentiform nuclei and thalamus. (B) T2, (C) susceptibility‐weighted, and (D) T1 sequences were unremarkable (images shown left to right). [file MDC3-12-S10-s001.docx]

Not So Smooth Sailing: *FIG4*-Related Disease is a Differential Diagnosis of Rapid Onset Dystonia-Parkinsonism

MJ Georgiades^1,2^ MBBS PhD, D Wilson^1^ FRACP PhD, MC Garcia^1,2^ MBBS FRACP, R Boland-Freitas^3^ FRACP PhD, H Morales-Briceño^1,2^ MD FRACP, N Mahant^1,2^ FRACP PhD, VSC Fung^1,2^ PhD FRACP, AJ Martin^1,3^ MBBS FRACP

# Supplementary Material


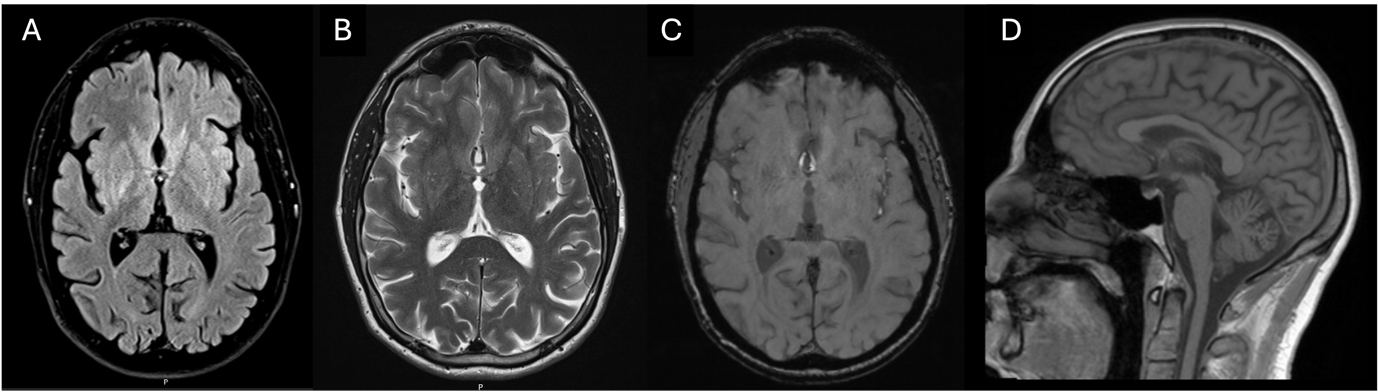


**Supplementary Figure 1**: A) Axial T2-FLAIR MRI of the brain showing asymmetric hyperintensity of the lentiform nuclei and thalamus. B) T2, C) susceptibility-weighted and D) T1 sequences were unremarkable (images shown left to right).
